# RETRACTION: Evaluation of SDS‐Coated Iron Nanostructure on the Gene Expression of Bio Surfactant‐Producing Genes by *Pseudomonas Aeruginosa*

**DOI:** 10.1002/elsc.70007

**Published:** 2025-02-26

## Abstract

**RETRACTION**: Y.A. Arani, Z. Noormohammadi, B. Rasekh, F. Yazdian, and H. Kazemi, “Evaluation of SDS‐Coated Iron Nanostructure on the Gene Expression of Bio Surfactant‐Producing Genes by Pseudomonas Aeruginosa,” *Engineering in Life Sciences* 22, no. 9 (2022): 584–593, https://doi.org/10.1002/elsc.202200002.

The above article, published online on 24 August 2022, in Wiley Online Library (http://onlinelibrary.wiley.com/), has been retracted by agreement between the journal Editors‐in‐Chief, An‐Ping Zeng and Ralf Takors; and Wiley Periodicals LLC. Following an investigation by the publisher, the parties have concluded that this article was accepted solely on the basis of a compromised peer review process. In addition, a third party informed the publisher that Figures 4 and 5 were reproduced from two articles published either previously or in the same year, and were used here in a different scientific context. The publisher has investigated and confirmed this, and found additional image manipulation in Figure 4. Therefore, the article must be retracted. Corresponding author Behnam Rasekh disagrees with this decision.

**RETRACTION**: Y.A. Arani, Z. Noormohammadi, B. Rasekh, F. Yazdian, and H. Kazemi, “Evaluation of SDS‐Coated Iron Nanostructure on the Gene Expression of Bio Surfactant‐Producing Genes by Pseudomonas Aeruginosa,” *Engineering in Life Sciences* 22, no. 9 (2022): 584–593, https://doi.org/10.1002/elsc.202200002.

The above article, published online on 24 August 2022, in Wiley Online Library (http://onlinelibrary.wiley.com/), has been retracted by agreement between the journal Editors‐in‐Chief, An‐Ping Zeng and Ralf Takors; and Wiley Periodicals LLC. Following an investigation by the publisher, the parties have concluded that this article was accepted solely on the basis of a compromised peer review process. In addition, a third party informed the publisher that Figures 4 and 5 were reproduced from two articles published either previously or in the same year, and were used here in a different scientific context. The publisher has investigated and confirmed this, and found additional image manipulation in Figure 4. Therefore, the article must be retracted. Corresponding author Behnam Rasekh disagrees with this decision.

